# Choosing between Higher and Lower Resolution Microarrays: do Pregnant Women Have Sufficient Knowledge to Make Informed Choices Consistent with their Attitude?

**DOI:** 10.1007/s10897-017-0124-5

**Published:** 2017-07-04

**Authors:** S. L. van der Steen, E. M. Bunnik, M. G. Polak, K. E. M. Diderich, J. Verhagen-Visser, L. C. P. Govaerts, M. Joosten, M. F. C. M. Knapen, A. T. J. I. Go, D. Van Opstal, M. I. Srebniak, R. J. H. Galjaard, A. Tibben, S. R. Riedijk

**Affiliations:** 1 Department of Clinical Genetics, Erasmus University Medical Center, Rotterdam, The Netherlands; 2 Department of Medical Ethics & Philosophy of Medicine, Erasmus University Medical Center, Rotterdam, The Netherlands; 3 Institute of Psychology, Erasmus University Rotterdam, Rotterdam, The Netherlands; 4 Department of Obstetrics and Prenatal Medicine, Erasmus Medical Centre, Rotterdam, The Netherlands; 5 Stichting Prenatale Screening Zuidwest Nederland, Rotterdam, The Netherlands; 6 Department of Clinical Genetics, Leiden University Medical Centre, Leiden, The Netherlands

**Keywords:** Prenatal diagnosis, Chromosomal microarray, Prenatal genetic counseling

## Abstract

Developments in prenatal testing allow the detection of more findings. SNP arrays in prenatal diagnosis (PND) can be analyzed at 0.5 Mb resolution detecting more clinically relevant anomalies, or at 5 Mb resolution. We investigated whether women had sufficient knowledge to make informed choices regarding the scope of their prenatal test that were consistent with their attitude. Pregnant women could choose between testing at 5 or at 0.5 Mb array. Consenting women (*N* = 69) received pre-test genetic counseling by phone and filled out the Measure of Informed Choice questionnaire designed for this study. Choices based on sufficient knowledge and consistent with attitude were considered informed. Sixty-two percent of the women made an adequately informed choice, based on sufficient knowledge and attitude-consistent with their choice of microarray resolution. Women who made an informed choice, opted for 0.5 Mb array resolution more often. There were no differences between women making adequately informed or less informed choices regarding level of experienced anxiety or doubts. Over time on T0 and T1, anxiety and doubts significantly decreased. While previous studies demonstrated that knowledge is an important component in informed decision-making, this study underlines that a consistent attitude might be equally important for decision-making. We advocate more focus on attitude-consistency and deliberation as compared to only a strong focus on knowledge.

## Introduction

Prenatal genetic screening and follow-up diagnostic testing confront pregnant women with often difficult decisions. One of the first decisions women make is whether or not to participate in prenatal screening. When deliberating whether or not to participate in prenatal screening programs, many women may find it difficult to understand the characteristics of the test, to weigh its benefits and risks and to grasp the possible implications (van Schendel et al. 2015).

The use of new, increasingly complex techniques, it is feared, may further hinder informed choices (de Jong et al. 2013, 2014; Dondorp et al. 2015). To date, there has been little empirical evidence to support or falsify the concern that women may no longer be able to make informed decisions regarding more complex prenatal tests. While techniques in prenatal screening and diagnosis are developing rapidly, the need for insight into whether pregnant women are making informed choices about prenatal genetic testing becomes ever more pressing.

The stated aim of prenatal screening is to offer reproductive options, allowing pregnant women to choose the best course of action if their unborn child is affected (Dondorp et al. 2015). These actions may include preparing for the future, altering pregnancy management or terminating a pregnancy. Prenatal screening and diagnosis should thus provide information about the fetus that is relevant to reproductive decision-making. Information that is not relevant to reproductive decision-making, it is argued, consequently falls outside the scope of prenatal screening (Dondorp et al. 2012). Information outside of this scope can be unwanted, for it may be burdensome and could lead to worry or anxiety for pregnant women. Moreover, such information may needlessly infringe upon their child’s right not to know its genetic risk (de Jong et al. 2014; Dondorp et al. 2012). Although the scope of prenatal screening should thus be limited to information that is relevant to reproductive decision-making, what is considered to be relevant is a topic for debate.

At present, in the Netherlands, prenatal screening is limited to detecting an increased risk of trisomies 13, 18 and 21. However, in our center we employ whole genome SNP arrays for prenatal diagnosis. One of the major consequences of using SNP array instead of more targeted techniques (such as rapid aneuploidy detection or conventional karyotyping) is that many more genetic aberrations may be detected (e.g., early onset diseases such as Williams syndrome, and Duchenne muscular dystrophy). Genetic aberrations may even include susceptibility loci (SL: 1.4%) (Van Opstal et al. 2015). SL are complicated test results because although they are defined as “likely pathogenic” (Srebniak et al. 2014), the associated risk of expression and severity is yet unquantifiable. SL are associated with neurodevelopmental disorders such as learning disabilities, behavioral problems and/or seizures (Srebniak et al. 2011; Van Opstal et al. 2015). We reported on the first parents’ experiences with prenatal disclosure of SL in a previous study (van der Steen et al. 2016). Outcomes like these may be equally relevant to reproductive decision-making. There is tension between the legal scope of prenatal screening in the Netherlands and its stated aim of enabling reproductive autonomy. There is also a tension between the scope of SNP array for follow-up diagnostic testing at our clinic, and the scope of screening in the national prenatal screening program, which is much narrower.

This contentious topic leads to much discussion amongst professionals and ethicists about which test to employ and what to report to pregnant women regarding prenatal genetic test outcomes. Some emphasize that test results which fall outside the scope of prenatal screening might put an unnecessary burden on pregnant couples (de Jong et al. 2014), while others argue that withholding any kind of information is paternalistic and should be avoided (McGillivray et al. 2012).

A prerequisite for reproductive autonomy is making an informed choice. Marteau et al. (2001) state that “An informed decision is one where all the available information about the health alternatives is weighed up and used to inform the final decision; the resulting choice should be consistent with the individual’s values. An effective decision is one that is informed, consistent with the decision-maker’s values and behaviorally implemented” (p. 100). Well-informed choices are psychologically beneficial (Kleinveld et al. 2009; van den Berg et al. 2006). Psychological management of prenatal test decisions is better when knowledge is adequate (Dahl et al. 2011), while uninformed choices increase decisional conflict and decrease feelings of personal wellbeing (Dahl et al. 2011). Psychological coping and informed choice were more difficult for pregnant women who were not prepared for the possibility of an abnormal prenatal screening result (Kleinveld et al. 2009). Studies reported that a majority of pregnant women did not make informed decisions regarding prenatal screening. And most women did not have sufficient knowledge to prepare them for the possibility of abnormal outcomes of prenatal screening (McCoyd 2013; Schoonen et al. 2011). Without adequate information provision and counseling, offering prenatal diagnosis with a wider scope could indeed burden the pregnant couple and undermine their reproductive autonomy instead of enhancing it. Making informed choices is meant to prevent the harms that too much unwanted information could cause.

What pregnant couples wish to learn about the health of their fetus is underreported thus far. The few studies on this subject indicate a preference among pregnant couples to learn as much as possible from prenatal diagnosis (PND) (Riedijk et al. 2014; van der Steen et al. 2014). We have recently reported that the vast majority of pregnant couples to whom we had offered the choice between array at higher (0.5 Mb) or lower resolution (5 Mb, comparable to CK), chose higher resolution array. In our experience, most pregnant couples at increased risk for common aneuploidies chose to learn as much as possible about the (future) health of their unborn child (van der Steen et al. 2014). We furthermore offered couples a choice whether they wished to be informed of SL if detected. Eighty-four percent of the pregnant couples engaging in PND chose to be informed of SL should these be detected (van der Steen et al. 2014). Using SNP arrays as a diagnostic prenatal test leads to the poignant question of the extent to which pregnant couples have sufficient knowledge to make informed decisions regarding its scope (van der Steen et al. 2014).

In this study we report on one member of pregnant couples, that is, pregnant women at increased risk for common aneuploidies who were offered a choice between 0.5 and 5 Mb SNP array testing. We investigated whether they had sufficient knowledge to make an informed decision consistent with their attitude. Furthermore, we explored whether level of informed choice was associated with anxiety and doubts.

## Materials and Methods

### Participants

**Fig. 1:**
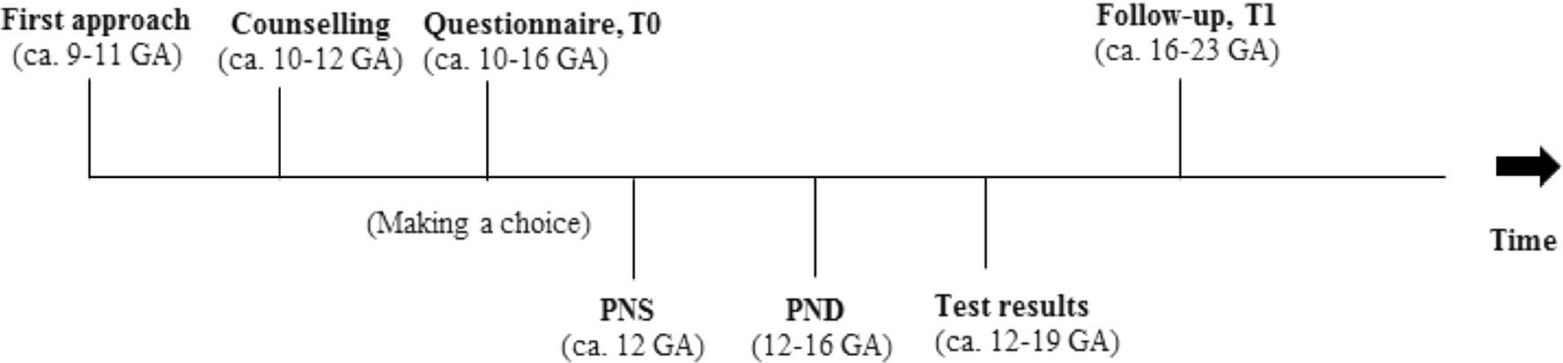
Timeline of the study

Pregnant women (*N* = 69) consented to participate from February 2012 to September 2013 at our outpatient prenatal clinic. Inclusion criteria were: a) advanced maternal age (>36 years), and/or b) the woman participated in first-trimester prenatal screening (PNS) or PND, and c) fluency in Dutch language. Women were approached at the intake of their first ultrasound, around 9–11 weeks gestational age (GA) and counseled by a clinical geneticist (see Fig. 1 for a timeline of the study). After counseling, women filled out a questionnaire about their choice. The Measure of Informed Choice, see Measures section, was filled out by a subsample of women that participated in our previous study (van der Steen et al. 2014).

### Procedure

This study was waivered by the local Medical Ethical Testing Committee (METC). An information leaflet about the study was added to the invitation letter pregnant women received before attending the outpatient clinic. A research-assistant was present at the clinic to approach pregnant women meeting the inclusion criteria and provide information concerning the study and its further procedures. After consenting, an additional genetic counseling session with a clinical geneticist by telephone was planned in advance of the next appointment for PNS or PND. We combined women engaging in PNS (hypothetical choice) and PND (real choice) in our sample to obtain a larger number of participants. Face-to-face counseling was not practically feasible in this study.

Women were approached at the intake around 9–11 weeks gestational age (GA) and counselled by a clinical geneticist, after which they filled out a questionnaire (T0) (see Fig. 1). Between 16 and 23 weeks GA, women were approached for follow-up by phone. This was four weeks after their prenatal test results (T1).

#### Content of Genetic Counseling by Telephone

Participating women received a 30–45 min counseling from a clinical geneticist (or a resident) by phone. Extensive information was provided. In addition to the background of genetics, participants were informed of the difference between 5 Mb and 0.5 Mb array. The 5 Mb array was presented as a “less broad test,” and it was specified that trisomy 13, 18 and 21 and other microscopically visible deviations could be found, comparable to the scope of a karyotype. The 0.5 Mb array was presented as a “broader test,” and examples of what could be detected additionally by broader testing compared to less broad testing was illustrated with Williams syndrome, Duchenne muscular dystrophy, and susceptibility loci for neurodevelopmental disorders (SL); these were counseled as incidental findings. Initially, participants could choose between 5 Mb testing and 0.5 Mb testing. The 0.5 Mb array resolution was presented as a broader test that also included susceptibility loci. During data collection, an increasing number of participants wished to learn the results of 0.5 Mb resolution array, but without disclosure of susceptibility loci. Therefore, we adopted the policy that participants could also opt for 0.5 Mb (broader testing) without being informed of susceptibility loci. The last part of the counseling comprised a dialogue about the women’s concerns, attitudes towards the scope of testing, questions and preferences. Women were asked whether they already knew what resolution they would choose. If necessary, additional information or explanation was provided.

### Measures

#### Demographics

Socio-demographic data were collected (living situation, educational level, nationality, religion, and age).

#### Measure of Informed Choice

To explore informed decision making regarding the scope of PND, we developed the Measure of Informed Choice (MIC). The MIC is based on the Multi-dimensional Measure of Informed Choice instrument [MMIC, Knowledge Scale (α = .68), and Attitude Scale (α = .78)] by Michie et al. (2002), which measures knowledge and attitude towards PNS. Our MIC contains 7 items measuring knowledge (see Table 2) and 6 attitude items regarding the scope of PND (see Table 3). A decision was considered to be adequately informed if it was based on sufficient knowledge and if the decision was consistent with the attitude towards testing with higher or lower resolution array. The knowledge scale comprised multiple-choice items, and we determined a cut-off score of 5 or more correct answers to qualify as “adequate knowledge” (see Table 2). We used a very strict criterion because the choice we offered is controversial, and we wanted to maintain a high standard to evaluate our counseling. Michie et al. used a midpoint score (4.5) on 8 knowledge questions for knowledge to be qualified as sufficient. Thus, our criterion for “sufficient knowledge” is stricter. This should be taken into account when interpreting our results.

We developed the MIC questions based on the content of the counseling participants received. During counseling, there was a strong emphasis on explaining what the differences between 0.5 Mb and 5 Mb testing were, and what the respective scopes might and might not detect, with realistic examples of certain conditions. A team of clinical geneticists, psychologists and a statistician were involved with the development of the questions. The attitude scale comprised six statements with a 10-point response format and ranging from 1 (useless/not important) to 10 (very useful/very important) (see Table 3). A higher score indicated a more positive attitude towards broader scope array (0.5 Mb), a lower score indicated a more negative attitude. Based on design of the MMIC from Marteau et al. (2001), we created three categories of outcomes of informed choice; 1) completely informed (adequate knowledge and consistent attitude), 2) partly uninformed (poor knowledge and consistent attitude, or good knowledge and inconsistent attitude) and 3) completely uninformed (poor knowledge and inconsistent attitude), (see Fig. 3).

#### Decisional Ambivalence Scale

The questionnaire furthermore comprised the previously published Decisional Ambivalence Scale (DAS; (Cronbach’s α = .85) (van der Steen et al. 2014). The DAS contain ten items that measure doubts and confidence regarding the choice. All items had a 10-point response format and ranged from 1 (not at all) to 10 (very much so). Summed scores on the DAS can range from 10 to 100, with a higher score indicating a higher level of experienced doubts.

#### STAI

Anxiety was measured using the short version of the Dutch state trait anxiety inventory (STAI), which was validated for pregnant women in the Netherlands(van der Bij et al. 2003). The scores ranged from 1 (not at all) to 4 (very much so). STAI total scores can range from 20 to 80. Higher scores indicate greater feelings of anxiety (van der Steen et al. 2014).

### Statistical Analyses

To obtain a larger sample, women engaging in PNS (hypothetical choice) and PND (real choice) were both included in our analyses. It should be noted that these are two different groups of women, and that the PND group is a “high stakes” group compared to the PNS group, that has lower stakes. Women in the PND group had made a real choice that led to real prenatal test results, and therefore they could have, arguably, paid more attention to the counseling. However, as there were no statistically significant differences in informed choice between the two groups of women, we analyzed the sample as a whole.

Before analyzing the data for this study, assumptions for ANOVA were checked. A significance level of *p* < 0.05 was used for all analyses. Outliers were detected, reverse-scored items were recoded, and total scores were calculated. To examine the internal consistency of the MIC, Cronbach’s alpha was used.

To assess whether participants made an informed choice, we calculated total MIC Knowledge and Attitude scores. Correct answers on the Knowledge scale were coded into dichotomous scores (1 = correct; 0 = incorrect) 1, thus summed to a maximum of 7 points. For the Attitude scale, with six statements, scores ranged from 6 (very negative) to 60 (very positive). Those were summed and divided by 6 to produce an attitude score between 1 and 10. Similar to other studies on this subject (Dahl et al. 2011; Kleinveld et al. 2009; Michie et al. 2002), we employed a midpoint score for the attitude scale; participants with an attitude score below 5.5 were categorized as having a negative attitude, scores above 5.5 were categorized as a positive attitude. Attitudes were checked for their congruence with the choice of array resolution. For example, if a participant indicated that knowledge about SL was important/useful, 0.5 Mb array resolution including disclosure of SL was expected as a choice. Attitudes were linked to choice of test for (in)consistency.

To examine the relationship between nominal variables, separate Pearson chi-square tests were used for decision outcome and actual (PND) and hypothetical (PNS) choice, decision outcome and broad (0.5 Mb) or less broad (5 Mb) array, and for decision outcome and wanting to be informed about SL (+SL/−SL).

We assessed differences in background variables (age, level of education) for women making an informed vs. an uninformed choice using separate Pearson chi-square tests. Using the decision outcome (completely informed/uninformed) as dichotomous factors, we performed separate independent samples t-tests for continuous variables (STAI/DAS total scores) to test for differences between groups. To assess differences in anxiety and doubts (DAS/STAI) between women opting for or against disclosure of SL, independent t-tests were performed. For anxiety and doubts over time (T0 & T1), we used paired samples t-tests.

## Results

### Demographic Variables

The mean age of women was 37.9 years. The demographic variables (see Table 1) of women making informed or uninformed choices did not differ significantly, although the relationship between educational level and informed/uninformed choices was marginally significant (*p* = 0.055).

**Table 1:** Demographic characteristics of women who made informed versus uninformed choices (*N* = 69)

	Total	Informed	Uninformed	*p(*χ^2^) *
	N (%)	N (%)	N (%)	
Previous children				
Yes	42 (60)	25 (66)	17 (53)	.28
No	28 (40)	13 (34)	15 (47)	
Education				
Low-intermediate	25 (35)	12 (29)	12 (46)	.055
High	44 (65)	30 (71)	14 (54)	
Nationality				
Dutch	64 (93)	38 (88)	26 (100)	.07
Other	5 (7)	5 (12)		
Religious				
Yes	17 (25)	11 (26)	6 (23)	.83
No	51 (75)	31 (74)	20 (77)	
Test type				
PNS	39 (56)	22 (51)	17 (65)	.25
PND	30 (44)	21 (49)	9 (35)	

*2-sided Chi square tests performed.

### Measure of Informed Choice

Tables 2 and 3 present the items and descriptives of the MIC knowledge and attitude scales. The internal consistency reliability of the MIC Knowledge scale was α = .55, which is insufficient. This was caused by the fact that most women answered the questions with the same answers, resulting in lower variances, which led to a lower Cronbach’s alpha. The MIC Attitude scale had a reliability of α = .78.

**Table 2:** Item descriptives of MIC knowledge scale, 7 items (Cronbach’s. α = .55, *N* = 69)

Item (multiple choice)	Incorrect/correct	M	SD
Q1. Which conditions can be excluded by CVS or AC?	0/1	.78	.42
Q2. What is the risk of having a miscarriage?	0/1	.82	.39
Q3. Which conditions may the less broad test detect?	0/1	.50	.50
Q4. Which conditions are not detectable with the less broad test?	0/1	.79	.41
Q5. Which conditions may the broad test detect?	0/1	.88	.33
Q6. What is a susceptibility locus?	0/1	.53	.50
Q7. What could be the added value of the broad test for pregnant women?	0/1	.79	.41
Total knowledge score	0–7	4.78	2.88

**Table 3:** Item descriptives of MIC attitude scale(Cronbach’s. α = .78, *N* = 69)

Item	Range	*M*	*SD*
*1. For me, knowledge about Down syndrome is…*			
a. (1) Not of added value … (10) Useful	1–10	8.86	1.64
b. (1) Unimportant … (10) Important	1–10	9.08	1.51
*2. For me, knowledge about a small, but severe chromosomal abnormality is…*			
a. (1) Not of added value … (10) Useful	1–10	9.00	1.39
b. (1) Unimportant … (10) Important	1–10	9.04	1.46
*3. For me, knowledge about a susceptibility locus is…*			
a. (1) Not of added value … (10) Useful	1–10	6.45	3.29
b. (1) Unimportant … (10) Important	1–10	6.66	2.97
Total attitude score	6–60	47.90	11.1

**Fig. 2:**
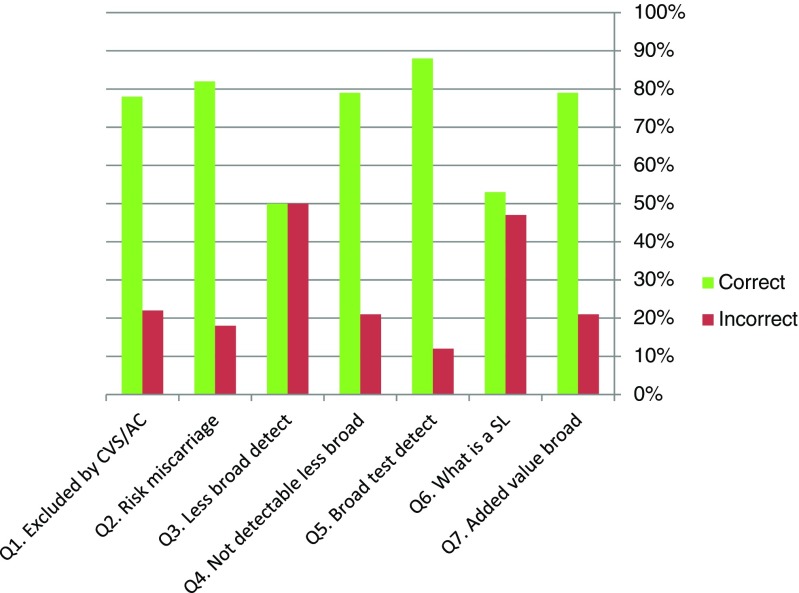
Percentage of women’s correct/incorrect answers on the MIC Knowledge scale (*N* = 69)

Figure 2 shows the percentage of correct and incorrect answers for the MIC Knowledge questions (see Table 2 for the specified questions). Most questions were answered correctly by the majority of women. Question 3, “Which diseases may the less broad test detect?” and question 6 “What is a susceptibility locus?” were answered correctly by approximately 50% of the women.

### Informed Choice Outcomes

**Fig. 3:**
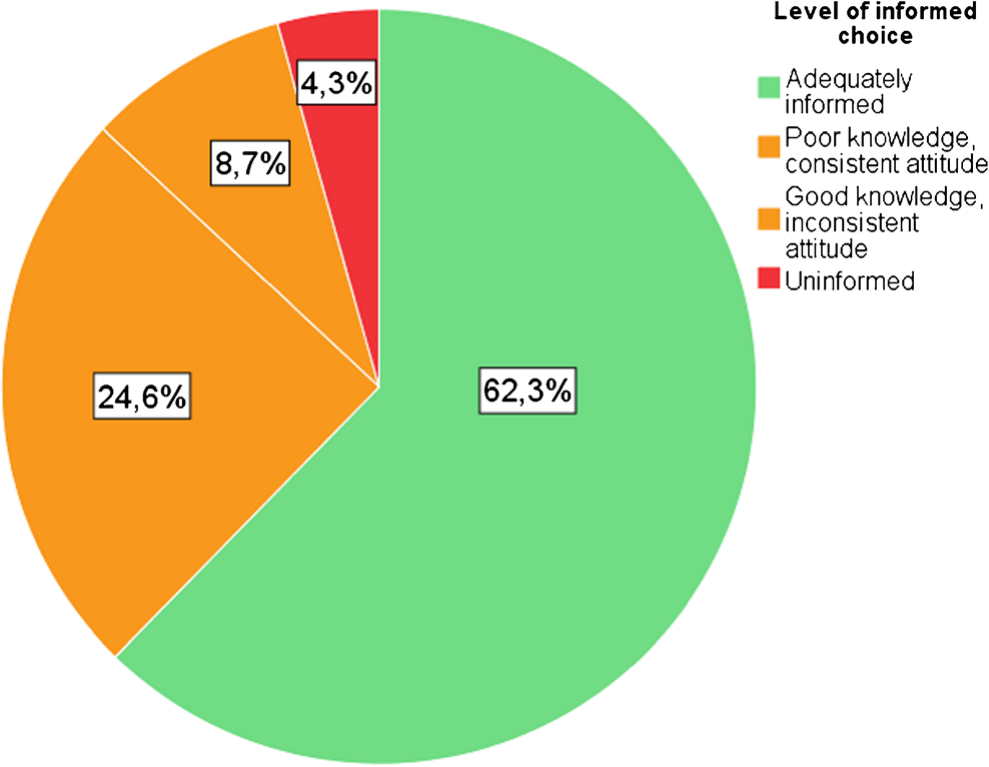
Pie chart of decision outcomes of all women (*N* = 69)

Figure 3 presents a pie chart of the outcomes of informed choice. Overall, 62.3% made a completely informed choice. A partly informed choice was made by 33.3% of women; 24.6% had poor knowledge, but a consistent attitude, and 8.7% had good knowledge, but an inconsistent attitude. Lastly, 4.3% made a completely uninformed choice.

For statistical analyses, level of informed choice was dichotomized in two levels, informed and uninformed.

### Relationship between Decision Outcome and Demographic Variables

There was a marginally significant association between educational level and informed/uninformed choices *(p* = 0.055). Women who had a higher educational level tended to be more likely to make completely informed choices than women who had a lower educational level. There were no other statistically significant differences in demographic variables (see Table 2).

### Relationship between Decision Outcome and Actual/Hypothetical Choices

As mentioned earlier, there was no significant association between informed/uninformed choices and actual versus hypothetical choice. Thus, women choosing for PNS or PND made equally informed choices.

### Relationship between Decision Outcome and Choosing Broader or less Broad Testing

There was a significant association between informed/uninformed choices and choice of array resolution χ^2^ (1) = 19.29, *p* < 0.001, *V* = 0.71 (large effect size), OR = 0.36 (95% CI 0.17–0.79). Women who made a completely informed choice, opted for broader testing more often.

### Relationship between Decision Outcome and Disclosure of SL

There was no significant association between wanting to be informed about SL and informed/uninformed choices. Thus, women opting for or against disclosure of SL made equally informed choices. There were no significant effects of wanting to be informed of SL on either anxiety or doubts.

### Anxiety & Doubts and Decision Outcome

There were no significant differences in anxiety level (STAI) and informed versus uninformed choices. There was no significant difference in levels of doubts (DAS) and informed versus uninformed choices. Thus, there were no differences in level of anxiety and doubts between women making informed versus uninformed choices. Mean anxiety and doubt scores for all women are displayed in Table 4.

**Table 4:** Mean anxiety (STAI) and doubts (DAS) score by decision outcome for all women (*N* = 69)

	Informed choice	Uninformed choice	*t*	*p*
	*n* = 37; 51%	*n* = 32; 58%		
Mean STAI score	34.05	34.95	n.s.	
Mean DAS score	25.11	25.81	n.s.	

There was a significant difference in anxiety (STAI) for women opting for PNS versus PND. Women who opted for PND, had a higher level of anxiety. There was no significant difference in level of doubt for women opting for PNS versus PND (see Table 5). There were no differences in anxiety and doubts between women opting for or against disclosure of susceptibility loci (see Table 6).

**Table 5:** Mean anxiety (STAI) and doubts (DAS) score of women opting for PND/PNS (*N* = 69)

	PND	PNS	*t*	*p*
	*n* = 29	*n* = 40		
Mean STAI score	37.20	33.75	6.802	.012
Mean DAS score	22.90	26.86	n.s.

**Table 6:** Mean anxiety (STAI) and doubts (DAS) score of women opting for or against disclosure of susceptibility loci (SL) (*N* = 62)

	+SL	-SL	*t*
	*n* = 39	*n* = 23	
Mean STAI score	36.49	32.22	n.s.
Mean DAS score	22.73	24.10	n.s.

Overall the anxiety and doubt scores decreased significantly between T1 and T2 (see Table 7). There were no significant differences in the course of anxiety and doubts between informed and uninformed decision-makers or between the PND and PNS subgroups.

**Table 7:** Mean anxiety (STAI) and doubts (DAS) score of women after counseling (T0) and 4 weeks after disclosure of prenatal test results (T1) (N = 69)

	T0	T1	*t*	*p*
	*n* = 69	*n* = 69		
Mean STAI score	35.03	26.82	5.390	<.001
Mean DAS score	23.86	18.32	5.134	<.001

## Discussion

The aim of the current study was to assess whether pregnant women had sufficient knowledge to make an informed decision regarding the scope of their invasive prenatal genetic test using SNP microarray that was consistent with their attitude. Furthermore, we explored whether level of informed choice was associated with anxiety and doubts.

Informed choice implies making a value-consistent decision based on sufficient knowledge. Although the majority of women made a “completely informed” choice (sufficient knowledge and consistent attitude), a substantial subgroup made a choice that was at odds with their personal values. Michie et al. (2002) showed that knowledge plays no role in whether women undergo screening or whether they act in line with their attitudes. Our study supports this finding; women who did not have sufficient knowledge were able to make a choice that was consistent with their attitudes, and vice versa. A small percentage of women with sufficient knowledge were still choosing value-inconsistently.

Anxiety and doubts were not higher in women who made an uninformed choice. These findings are in contrast to previous studies showing that making uninformed choices is associated with adverse psychological outcomes. Women who made uninformed choices had more decisional conflicts/doubts and felt more anxious when making a choice whether or not to engage in prenatal screening (Dahl et al. 2011; van den Berg et al. 2006). Therefore, we would have expected that making an uninformed choice (lack of knowledge) might bring more worries about choosing the right test, and that these worries may lead to more anxiety or stress. Alternatively, it is possible that the women who made an uninformed choice were not able to grasp the potential detrimental consequences of their choice. A lack of knowledge may only be stressful if one is aware of what is lacking. The percentage of informed choices are concordant with other studies on informed choice in prenatal *screening,* although those studies used less stringent criteria, such as the midpoint score (Michie et al. 2002; Rowe et al. 2006; Schoonen et al. 2012).

The concepts of adequately informed and uninformed choices are evident. However, partly informed choices lie in a more “grey area,” which needs to be reflected upon. We found that most women who made partly informed choices based their choice on insufficient knowledge but with a consistent attitude. It could be argued that partly informed choices can still be considered autonomous choices: women may not need the complete detailed facts and specifications about the test characteristics to make a choice that is in line with their values (or an expression of self-determination). In line with this, it may be argued that reproduction/recall of knowledge after counseling is less of a condition for autonomous choice than agreement with one’s personal values.

Choices based on sufficient knowledge, but with an inconsistent attitude, were less prevalent. In line with an earlier study, our results show that knowledge indeed played no role in whether or not the women acted in accordance with their attitudes (Michie et al., 2003). We argue that value-inconsistent choices might be the most worrisome type of decision. If a woman chooses a scope of testing that does not fit her personal values, despite having sufficient knowledge about the test characteristics, this might lead to adverse psychological outcomes. It has to be taken into account that subtle signs of attitude inconsistency are easily missed. The counselors’ preferences may have (inadvertently) influenced the pregnant women’s decision-making, leading women to make choices that were inconsistent with their own values despite enough knowledge.

### Actual versus Hypothetical Choices

No differences were found in the level of informed choice between the PNS and PND groups. This might indicate that women who made a hypothetical choice, have gone through a similar decision (−making) process as women who underwent PND. However, there is a difference between the women engaging in PNS and PND: women engaging in PND made a real choice that was actually performed by our lab, and thus had higher stakes than women making a hypothetical choice (PNS). Our results show that women opting for PND indeed experienced more anxiety than women engaging in PNS. It might be that level of anxiety is associated with informational needs. Alternatively, it could be that women engaging in PND are more anxious because of the miscarriage risk associated with the invasive procedure (Muller and Cameron 2014). It must be noted that in our sample 19% of the pregnant women experienced anxiety at clinically relevant levels, and these women were distributed equally across the PNS/PND groups. At follow-up, four weeks later and after they received test results, almost all anxiety scores were back to normal levels (van der Bij et al. 2003), except for three women who experienced enduring anxiety.

### Broad or less Broad Microarray and Susceptibility Loci

Women who opted for broad scope PND made informed choices significantly more often. Being fully aware of the possible outcomes, they preferred to gain information about susceptibility loci in their unborn child. This might be related to the “sense of personal ownership” of genomic data (Kimball et al. 2014). Patients may be inclined to want ownership and/or control over their – or in this case their baby’s - genomic data. This may contribute to choosing a maximum of information from a genomic test, even if that means the test would include uncertain outcomes such as susceptibility loci (van der Steen et al. 2014). Our first impressions were that women in our clinic appeared to be able to handle this kind of information (van der Steen et al. 2016). Moreover, women indicated that they could use this kind of information in the future, if their child would develop abnormally. They indicated that they would know where to start looking for help and/or mobilize adequate care.

The majority of well-informed women chose to be informed of susceptibility loci if detected. The finding that the majority of women make informed decisions about susceptibility loci might seems in contrast with the often expressed fear/concern among professionals that these results are too difficult to grasp for patients (de Jong et al. 2009; de Jong et al. 2013). However, the two positions are not mutually exclusive. Women may be able to make an informed decision at the time, but not able to fully grasp/understand the long-term consequences of a rather abstract test outcome. On the other hand, professionals might underestimate the resiliency of their patients. We found that the women in our study did not have a heightened level of anxiety compared to other Dutch pregnant women at high risk of an abnormal fetus (van der Bij et al. 2003). This finding is also incongruent with professionals’ worries of burdening pregnant women with an overload of information (de Jong et al. 2009; de Jong et al. 2013; Dondorp et al. 2012; McGillivray et al. 2012).

In conclusion, despite all of the controversy regarding prenatal microarrays, our study shows that the majority of women were capable of making an informed choice regarding the scope of their invasive prenatal genetic test. And most importantly, they made informed choices in the absence of severe anxiety or doubts. Our data have shown that overall levels of anxiety and doubts decreased significantly over time, regardless of the choices (broad, less broad, SL or no SL) or level of informed decision-making. This decreasing pattern of anxiety is in accordance with previous studies (van der Bij et al. 2003).

It should be noted that choice/consent cannot and need not be *completely* informed (Manson and O'neill 2007). People may differ with regard to their informational needs and the level of detail they require for decision-making (Vos et al. 2013). For some, knowing that testing may generate “information about severe, incurable conditions” may be sufficient, whereas others may need to know what conditions exactly are included in the test, in order to make an informed decision. To accommodate differences in informational needs among individual women, pre-test counseling can be conducted in a layered fashion, where basic, crucial information is offered to all women, and further, more detailed information is given if needed or desired (Bunnik et al. 2013). The level of knowledge required for informed choice, it can be argued, may thus vary among individual decision-makers. Attitude consistency, on the other hand, is less of a spectrum, but rather a necessary condition for informed choice.

In the literature, efforts aimed at improving informed choices mostly target the knowledge component (Schoonen et al. 2011; Schoonen et al. 2011; Schoonen et al. 2012). We stress the importance of attitude consistency, and recommend that the choices of pregnant women regarding the scope of their genetic test should fit their personal values well in order to facilitate informed choice. Thus, interventions aimed at improving informed choices through attitude consistency may be more effective than those targeting knowledge only (Michie et al. 2002; van den Berg et al. 2006). We suggest that attitude and values need to be explored and discussed in the pre-test counseling sessions.

The telephone counseling enabled the majority of women to make an informed choice. It must be taken into account, however, that this counseling was extensive and time-consuming, and therefore will not be feasible in everyday practice. It would be interesting to compare the level of informed choices with telephonic versus routine face-to-face counseling. Face-to-face counseling has a more personal aspect, and therefore might be capable of more adequately addressing attitude inconsistency or miscomprehension.

### The Future: The Expansion of Prenatal Genetic Information

Prenatal screening and follow-up diagnostic testing are likely to expand in the future, and to become more complex, as more and more findings/conditions could be included in the test. This might not only be possible with invasive testing, but also with non-invasive prenatal test (NIPT). Some state that in order to keep informed consent feasible, unnecessary complications should be avoided: screening should only be used for trisomies 13, 18 and 21. However, complications may not always be unnecessary: tests may come to include other conditions that are *as relevant* to women or couples in reproductive decision-making as is Down syndrome. Multiple studies have shown that a majority of pregnant women prefer an individualized choice, and prefer to learn as much as possible from prenatal tests (de Jong et al. 2013; van der Steen et al. 2014; van Schendel et al. 2015). Broadening the scope of prenatal diagnosis should - at minimum - be considered. To facilitate informed choices, pre-test counseling remains of great importance. Since extensive face-to-face counseling might not always be feasible, the next step may be to develop decision-aids that comprise both knowledge and attitude and personal values. This could be especially helpful for women who may otherwise make choices that are inconsistent with their attitudes. Such solutions and new models of informed consent are more and more widely applied in healthcare systems, and they may indeed have the potential to improve complex decision-making regarding the prenatal screening and follow-up diagnostic testing offer (Vlemmix et al. 2013).

### Strengths & Limitations

It should be noted that the women who present at the prenatal clinic of our university medical center are of above-average educational level. Furthermore, participating women were already motivated to seek prenatal screening or diagnosis (on the basis of either advanced maternal age or abnormal first trimester screening), and may thus be more inclined to prefer to learn about genetic risks in their fetuses than other pregnant women. Our results might be further biased due to the unequal distribution of ethnicity; only 7% of the women was not Dutch.

A strength of this study is that, to our knowledge, we are the first to have assessed informed choice regarding invasive PND performed with microarrays. Further, we allowed participants to make an individualized choice of array resolution that best suited their preferences.

## Conclusion

We found that the majority of pregnant women were capable of making an adequately informed choice about the scope of invasive PND, including whether or not they wanted to be informed of SL. A justified course of action based on this result could be that laboratories perform broad analysis and counselors provides patients with an opting in or out possibility. Knowledge has already been established as an important component in informed choice. However, our study underlines that a consistent attitude might be equally important. We anticipate that in the future, regardless of more complex or new techniques, the majority of women will still be able to make informed choices, as long as adequate information provision and counseling are provided. For counseling practices, we advocate a stronger focus on attitude-consistency instead of only a focus on knowledge.
